# Hierarchically Porous Polyaniline Exhibiting Enhanced Pseudocapacitive Property from Copolymerization of Aniline and Tetrakis(4-aminophenyl)methane

**DOI:** 10.3390/polym17223062

**Published:** 2025-11-19

**Authors:** Jinsoon Choi, Kyeong Eun Yeo, Ji-Woong Park

**Affiliations:** Department of Materials Science and Engineering, Gwangju Institute of Science and Technology (GIST), 123 Cheomdangwagiro, Gwangju 61005, Republic of Korea; cjs890221@gm.gist.ac.kr (J.C.); keyeo1462@gm.gist.ac.kr (K.E.Y.)

**Keywords:** polyaniline, hierarchical porosity, supercapacitor, oxidative polymerization, conducting polymers

## Abstract

Hierarchically porous polyaniline (PANI) was synthesized by oxidative copolymerization of tetrakis(4-aminophenyl)methane (TA) and aniline. Mixing TA with ammonium persulfate (APS) followed by aniline addition generated both tetra-arm star-shaped and linear PANIs; their insolubility in the reaction medium led to aggregation into solid precipitates. During assembly, linear PANI formed nanofibers, while star-shaped PANI created short branches on the nanofiber surfaces. The TA molar fraction in the feed governed morphology and properties: higher TA increased specific surface area and the mesopore fraction but decreased electrical conductivity. Balancing this porosity–conductivity trade-off identified PANI (TA 3%) as optimal, exhibiting the highest CV current response with pseudocapacitive profiles, with a specific capacitance of 556 F g^−1^ versus 380 F g^−1^ for pristine PANI. Device-level galvanostatic charge–discharge yielded 239 F g^−1^ at 0.1 A g^−1^ (vs. 199 F g^−1^), while high-rate performance was limited by conductivity. These results show that introducing a small comonomer fraction to promote star-chain growth can produce hierarchical porosity and enhance pseudocapacitive behavior; further conductivity enhancement is expected to improve high-rate capacitance.

## 1. Introduction

Supercapacitors, particularly pseudocapacitors, store energy through rapid and reversible faradaic reactions, which enable them to achieve high power density and exceptional cycle life. Among various pseudocapacitive materials, conducting polymers (CPs) such as polyaniline (PANI) [1,2,3], polypyrrole (PPy) [4,5,6], and poly(3,4-ethylenedioxythiophene) (PEDOT) [7,8,9] have been extensively explored due to their intrinsic electronic conductivity, mechanical flexibility, and multiple redox-active states. Their faradaic behavior allows for significantly higher specific capacitance than electric double-layer capacitors (EDLCs), positioning CPs as attractive candidates for next-generation energy storage systems [10].

Despite their promising redox properties, the electrochemical performance of conducting polymer-based electrodes cannot be predicted based solely on their electronic transport characteristics. Instead, it is critically governed by ion accessibility to electroactive sites, which is intrinsically linked to the specific surface area and internal pore architecture. To address this, hierarchical pore structures have emerged as an effective design strategy [11,12,13]. In these systems, micropores provide charge storage sites, while macropores act as transport highways for ion diffusion, effectively minimizing resistance and enhancing rate capability.

Traditional methods for adding porosity to conducting polymers—like template-directed synthesis [14], surfactant-assisted assembly [15], and interfacial polymerization [16]—produce nanostructured materials that enhance surface area and ion transport. However, each has drawbacks: template synthesis requires extra steps for template removal, risking residue; surfactant methods can leave behind surfactants that reduce conductivity and stability; and interfacial polymerization is limited by interfacial stability, affecting reproducibility and scalability. Overall, these methods rarely modify the actual polymer growth mechanism, limiting molecular-level control over porosity and redox site accessibility. Therefore, there is a growing interest in approaches that enable precise tuning of polymer architectures to overcome these limitations.

Here, we present a synthetic approach wherein a comonomer capable of generating star-shaped conducting polymer chains is copolymerized with a conventional monomer that forms linear polymer chains. Specifically, tetrakis(4-aminophenyl) methane (TA) is copolymerized with aniline to produce hierarchically porous polyaniline (PANI). This methodology induces competitive propagation between linear and star-shaped polymer chains in the polymerization solution. The distinct aggregation behaviors of linear and star polymer chains lead to hierarchically porous PANI structures, whose properties are adjustable through comonomer composition.

While the incorporation of rigid TA units within PANI chains may reduce crystallinity and affect conductivity, an optimal TA content balances ion accessibility and electronic percolation. Notably, the PANI (TA 3%) composition shows enhanced capacitive performance, delivering a specific capacitance of 239 F g^−1^ at 0.1 A g^−1^. Unlike conventional strategies that rely on post-synthetic structuring, this template-free polymerization route offers intrinsic control over macromolecular architecture, enabling scalable synthesis of hierarchically porous conducting polymers with tunable pseudocapacitive properties.

The results of this study show that copolymerizing conventional monomers for conjugated/conducting polymers with a star-shaped comonomer modulates chain aggregation, producing new nanostructures without surfactants or templates. This comonomer-programmed, template-free route provides intrinsic control over chain topology and aggregate assembly, improving process simplicity, reproducibility, and scalability. By tuning composition, the strategy co-optimizes nanoscale architecture and electrochemical properties and is readily generalizable to other conducting polymers through judicious selection of star-initiating comonomers.

## 2. Materials and Methods

### 2.1. Materials

Aniline (≥99%, Sigma-Aldrich, St. Louis, MO, USA) was distilled under reduced pressure prior to use. Tetrakis(4-aminophenyl) methane (TA) was synthesized in our laboratory following a previously reported procedure. Ammonium persulfate (APS, ≥98%, TCI Chemicals, Tokyo, Japan) was used as received. Concentrated hydrochloric acid (36.5%, Sigma-Aldrich, St. Louis, MO, USA) was diluted with deionized water to obtain a 1 M HCl solution. *N*,*N*-Dimethylformamide (DMF, ≥99%, Sigma-Aldrich, St. Louis, MO, USA) was obtained anhydrous from a column-based solvent purification system.

### 2.2. Synthesis of PANI

Aniline (0.365 mL, 4 mmol) was dissolved in 1 M HCl (200 mL) in a 500 mL round-bottom flask and cooled in an ice bath (0–5 °C) under continuous stirring (340 rpm). In a separate vessel, ammonium persulfate (APS, 1.14 g, 5 mmol; 1.25 equiv relative to aniline) was dissolved in 1 M HCl (200 mL) and pre-cooled to 0–5 °C. The APS solution was added dropwise to the aniline (in 1 M HCl) while maintaining 0–5 °C, and the reaction was allowed to proceed for 24 h. The resulting dark-green precipitate was collected by vacuum filtration, washed thoroughly with methanol and deionized water until the filtrate was colorless, and dried in a vacuum oven at 60 °C for 24 h to afford pristine PANI powder.

### 2.3. Synthesis of PANI (TA x%)

In a typical synthesis, ammonium persulfate (APS, 1.14 g), totaling 5 mmol, 1.25 equiv. with respect to the total moles of aniline and TA, was dissolved in 200 mL of 1 M HCl in a 500 mL round-bottom flask equipped with mechanical stirring and placed in an ice bath (0–5 °C). Tetrakis (4-aminophenyl) methane (TA, x mol% relative to aniline, corresponding weight) was dissolved in 1 mL of DMF and added to the APS/HCl solution under stirring. The initially clear solution gradually turned yellow within 30 min, indicating the formation of TA radical cations.

Separately, aniline (0.365 mL, 4 mmol) was dissolved in 200 mL of 1 M HCl to prepare a stock solution. This aniline solution was introduced dropwise into the TA cation radical solution using a separatory funnel under continuous stirring at 340 rpm, while maintaining the reaction temperature at 0–5 °C. The polymerization was allowed to proceed for 24 h under these conditions. After completion, the resulting precipitate was collected by vacuum filtration, washed repeatedly with methanol and deionized water until the filtrate became colorless, and dried in a vacuum oven at 60 °C for 24 h. A dark-blue powder was obtained, which appeared slightly deeper in color compared to conventional polyaniline synthesized under identical conditions.

### 2.4. Electrode Ink Preparation

The electrode ink was prepared by dispersing the active material (0.0085 g), carbon black (0.0015 g), and Nafion solution (10 wt%, 22.8 μL) in ethanol (2.0 mL). The mixture was sonicated to obtain a homogeneous suspension. A 15 μL aliquot of the ink was drop-cast onto a glassy carbon disk working electrode (GCE) and dried at room temperature. The mass loading of active material was calculated from the ink composition and the deposited volume.

### 2.5. Electrode Preparation for Capacitance Measurement

Circular carbon paper disks were used as current collectors/substrates. The carbon paper was first treated with concentrated H_2_SO_4_ to enhance wettability, thoroughly rinsed with deionized water, and dried. An electrode ink was prepared by dispersing 0.01 g of PANI (TA 3%) in 1 mL of ethanol with 22.4 μL Nafion (10 wt%). A defined volume of the ink was drop-cast onto the carbon paper disk and dried at room temperature to obtain the target mass loading of active material. Two-electrode Swagelok-type cells were assembled using stainless-steel pistons/current collectors, a paper separator, and 1 M H_2_SO_4_ aqueous electrolyte. Ink-coated carbon paper disks served as electrodes (disk geometry).

### 2.6. Characterization

Morphologies of the samples were examined using a field-emission scanning electron microscope (FE-SEM, Hitachi S-4700, Tokyo, Japan) operated at an accelerating voltage of 10 kV. Prior to measurement, all samples were coated with a thin platinum layer using a low-vacuum sputter coater (45 s) to improve conductivity and prevent charging during imaging.

UV–Vis absorption spectra were recorded using a spectrophotometer (Lambda 20, PerkinElmer, Shelton, CT, USA). For monitoring the early-stage polymerization process, aliquots of the reaction mixture were taken and diluted 50-fold with 1 M HCl before measurement.

FT-IR spectra were collected on a PerkinElmer 2000 Series spectrometer. Each spectrum was recorded with 16 scans at a resolution of 4.0 cm^−1^ and a data interval of 0.2 cm^−1^.

Nitrogen adsorption–desorption isotherms were measured at 77 K using a volumetric adsorption analyzer (ASAP 2020, Micromeritics, Norcross, GA, USA). Prior to analysis, samples were degassed under vacuum at 373 K for at least 10 h. The specific surface area was determined from the adsorption isotherm using the Brunauer–Emmett–Teller (BET) method, and the pore size distribution was obtained from the desorption branch using the Barrett–Joyner–Halenda (BJH) model.

The electrical conductivity of the samples was measured using the standard four-point probe method (CMT-100S, Jandel Engineering Ltd., Leighton Buzzard, UK). Powder samples were compressed into pellets prior to measurement. Electrical resistance was recorded using a digital source meter (Keithley 2450, Keithley Instruments, Solon, OH, USA), and conductivity was calculated from the measured resistance values and pellet dimensions.

X-ray diffraction patterns were collected on a Rigaku diffractometer (Rint 2000, Tokyo, Japan) operated at 40 kV and 100 mA using Cu Kα radiation (λ = 1.5406 Å). Data were recorded over the 2θ range of 5–40° at a scan rate of 0.5° min^−1^. Powdered samples were gently pressed onto a flat sample holder prior to measurement.

Cyclic voltammetry was performed in 1 M H_2_SO_4_ using a three-electrode configuration with the ink-coated GCE as the working electrode, an Ag/AgCl (3 M KCl) reference electrode, and a carbon rod as the counter electrode. The scan rate was 0.01 V s^−1^, and the potential window was 0.5–1.1 V vs. Ag/AgCl. Specific capacitance values were normalized to the mass of the active material. Specific capacitance from CV was obtained by integrating the current over the potential window and normalizing by mass and scan rate (10 mV/s):

$$C_{s}=\frac{1}{m_{active}s∆V}\int_{V_{1}}^{V_{2}}I\left(V\right)dV$$

m_active_: mass of active material on the carbon paper; V_1_, V_2_: lower/upper potential limits (V, vs. Ag/AgCl); s: scan rate (V s^−1^)

Galvanostatic charge–discharge (GCD) was performed at prescribed current densities (e.g., 0.1 A g^−1^). Specific capacitance was calculated from discharge curves and normalized to the mass of active material on the disk electrode (s). For symmetric two-electrode calculations, the per-electrode capacitance was obtained from the following equation:

$$C_{s}=\frac{4I\Delta t}{m_{active}\Delta V}$$

I: applied discharge current (A); Δt: discharge time (s); m_active_: mass of active material on the working electrode; ΔV: effective voltage window

## 3. Results and Discussion

**Figure 1:**
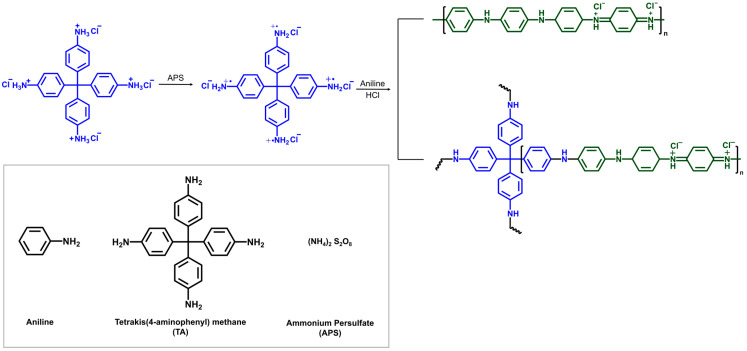
The polymerization yielding the mixture of linear and 4-arm star PANI chains. The blue structures represent the TA moiety within each polymer. TA was first added to a large excess of APS in 1 M HCl to form a TA radical cation solution, where the solution of aniline in 1 M HCl was added. The molar ratio of TA to aniline was adjusted to 0~10%.

To achieve a hierarchically porous PANI architecture, we employed a TA-driven copolymerization strategy. Figure 1 provides an overview of the synthetic process for producing the hierarchical structure of PANI. To generate star-shaped PANI chains, tetrakis(4-aminophenyl) methane (TA) was copolymerized with aniline, using ammonium persulfate (APS) as the oxidizing agent. The molar percentage of TA varied from 0 to 10%. TA was initially activated using excess APS, ensuring the growth of PANI chains from each of TA’s four arms when reacting with anilines. A TA solution in DMF was added to APS in 1 M HCl at 0–5 °C, producing a yellow solution of TA cation radical. Aniline in 1 M HCl was then added dropwise slowly to the solution of the TA cation radical. The sequential process facilitates the formation of tetra-arm PANI chains alongside the homopolymerization of anilines into linear PANI chains. The solution shifted from yellow to clear upon addition of aniline, then to a dark green viscous slurry over time. The resulting PANIs are labeled as PANI (TA x%), where x indicates the mol% of TA.

**Figure 2:**
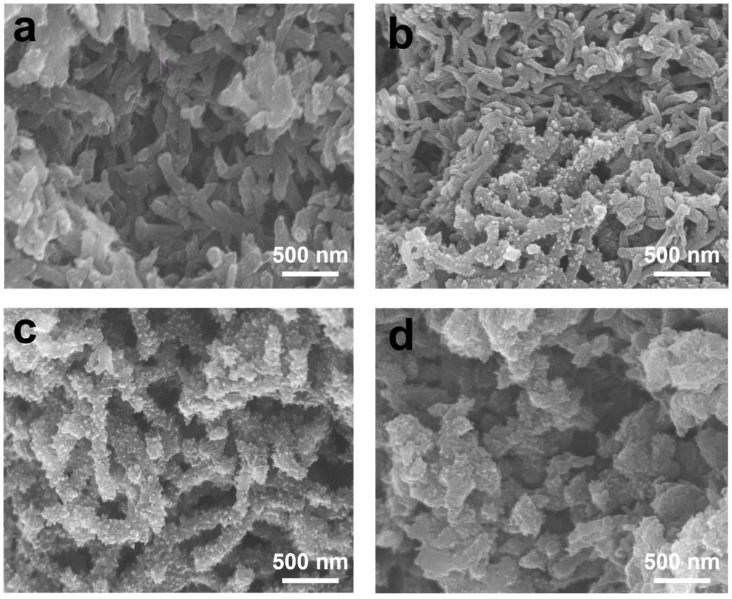
SEM images of polyaniline with or without TA (**a**) PANI; (**b**) PANI (TA3%); (**c**) PANI (TA5%); (**d**) PANI (TA10%).

Scanning electron microscopy (SEM, Tokyo, Japan) analysis shows morphological changes from PANI to TA-incorporated PANIs as TA loading increases (Figure 2). PANI displays a nanofiber morphology characteristic of oxidative polymerization (Figure 2a), with smooth, elongated fibers measuring approximately 40–50 nm in diameter. At 3 mol% TA (Figure 2b), both smooth and branched nanofibers are observed. When the TA concentration increases to 5 mol% (Figure 2c), all nanofibers exhibit rough, branched structures. The data suggests that introducing TA leads to the formation of short branches on the surfaces of the nanofibers originally derived from linear PANIs. At 10 mol% TA (Figure 2d), the nanofiber structure is less prominent, while the branched features remain present, resulting in an irregular granular morphology with branching characteristics.

The observation of branches on the PANI nanofiber surfaces suggests that the aggregation of star-shaped PANI chains with rigid tetrahedral geometry occurs after the initial nanofiber formation by linear PANI chains (Figure S2). Furthermore, it is probable that the rapid generation of nanofibers facilitates the nucleation and subsequent aggregation of these star-shaped chains on their surfaces. When the concentration of TA is high, the formation of nanofibers is hindered by the presence of rigid tetra-armed chains, ultimately leading to a rough, granular morphology.

**Figure 3:**
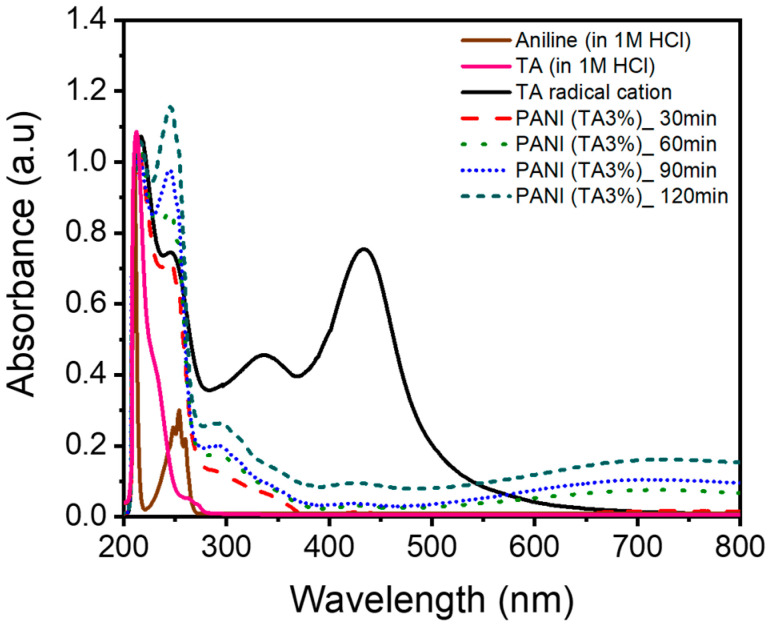
UV–Vis spectra of TA and aniline (in 1 M HCl); the TA radical cation formed upon APS addition; and the PANI (TA 3 mol%) recorded at 30, 60, 90, and 120 min after adding aniline in 1 M HCl to the TA/APS solution.

The polymerization of aniline in the presence of TA was monitored by time-resolved UV–Vis spectroscopy at 30 min intervals for up to 120 min, during which no precipitate was observed. Spectra for TA and TA/APS in 1 M HCl were also recorded as controls (Figure 3). The protonated TA (TA in 1 M HCl) displays an aromatic π-π * transition at ~212 nm, and the solution is colorless [17]. Upon addition of APS, TA is oxidized to the TA cation radical, introducing a singly occupied molecular orbital (SOMO) within the π-π * gap, thereby enabling optical transitions such as π-SOMO and SOMO-π* [18]. Spectroscopic evidence for TA cation radical formation includes a bathochromic shift of the π-π* band to approximately 217 nm with a vibronic shoulder at 245 nm [19], along with broad absorption peaks at 335 and 435 nm ascribed to the SOMO-involved transitions [20], resulting in a yellowish solution (Figure S3a).

The formation of the TA cation radical in the presence of excess APS and HCl enables the coupling of all four TA arms with subsequently added aniline monomers. Upon the addition of aniline (in 1 M HCl), the yellow coloration of the TA cation radical solution disappeared immediately (Figure S3b). Greenish colors appeared after stirring for over an hour. UV–Vis spectroscopy revealed benzenoid π–π * transitions at 246 nm [21], as well as polaron absorption bands in the 400–800 nm range [22], which increased in intensity over time. (Figure S3c,d).

Varying TA from 0 to 10 mol% did not measurably affect the precipitation induction period (~3 h under our conditions). Pre-generated TA cation radical signals were quenched rapidly upon aniline addition. Taken together, these observations suggest that the intrinsic reactivity of aniline toward oxidative polymerization is largely insensitive to TA within this range and that nanostructure formation begins once both star-shaped and linear chains attain molar masses sufficient for aggregation and precipitation. Consistent with this interpretation, the observed morphology–composition correlation indicates that the relative fraction of the star-shaped architecture varies systematically with TA content. Given the complexity of oxidative polymerization kinetics [23,24,25,26,27], a more detailed kinetic analysis may be of interest in future work.

**Figure 4:**
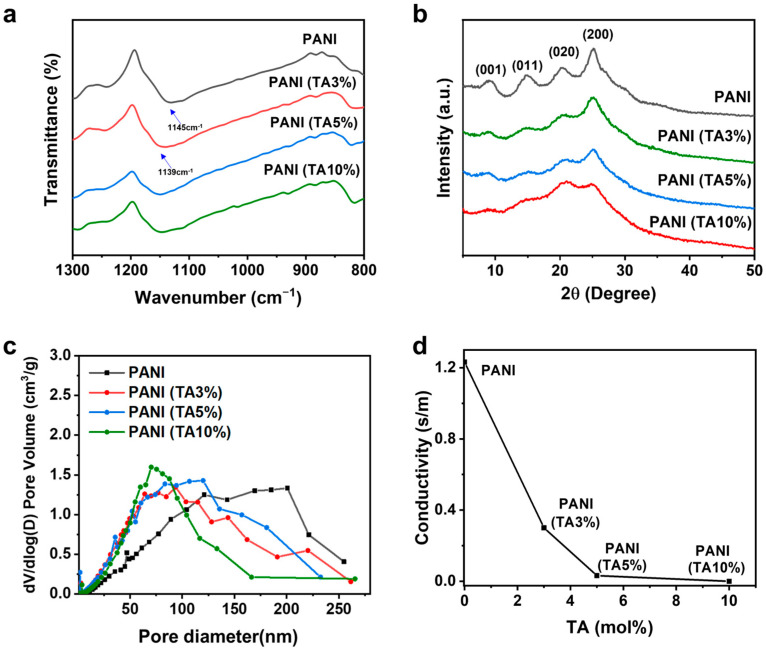
Structural characterization and electrical conductivity of PANI and PANI (TA x%). (**a**) Comparison of quinoid band position in the FT-IR spectra, (**b**) X-ray diffraction patterns, (**c**) BJH pore size distributions, and (**d**) electrical conductivity.

Comprehensive structural analyses were performed to correlate molecular structure with physical properties. In FT-IR spectra, the position of the characteristic quinoid stretching band varies with PANI conjugation length (Figure 4a) [28]. For TA-incorporated PANIs, this band shifts from 1139 to 1145 cm^−1^ as TA content increases, indicating a reduction in effective conjugation length. X-ray diffraction (Figure 4b) supports this conclusion: semi-crystalline PANI typically shows reflections at 2θ = 9.2°, 14.7°, 20.2°, and 25.0°, assigned to the (001), (011), (020), and (200) planes of emeraldine salt, respectively [29]. The (020) and (200) reflections report on interchain π–π stacking and parallel backbone periodicity [30], and both become weakened and broadened upon TA incorporation, consistent with reduced chain ordering/packing and shortened conjugation.

As shown by the SEM images (Figure 2), TA-containing chains increase the surface roughness of growing PANI nanofibers. BJH analysis (Figure 4c) shows that TA incorporation enhances porosity, shifting the pore size distribution toward the mesopore range (2–50 nm) and increasing BET surface area from 45 m^2^ g^−1^ for PANI to 95 m^2^ g^−1^ for PANI (TA 5%). Notably, a small fraction of TA comonomer substantially raises the mesopore fraction, imparting hierarchical porosity.

While TA incorporation enhances the porosity and mesopore content of the PANIs, electrical conductivity decreases with TA content (Figure 4d). The diminished conductivity is attributable to poorer interchain packing and shorter conjugation lengths, as evidenced by the IR and XRD results.

**Figure 5:**
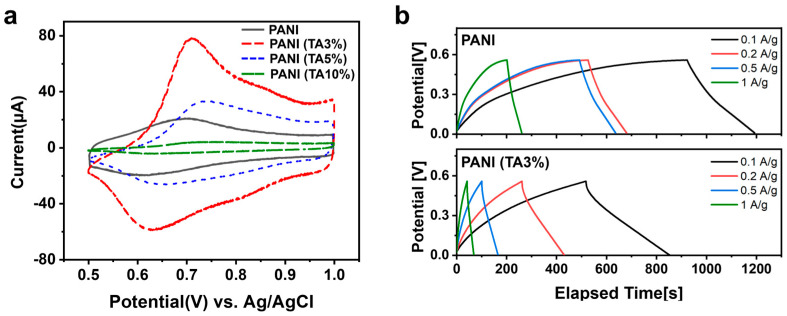
Electrochemical performance of PANIs. (**a**) Cyclic voltammograms of pristine PANI and PANI (TA x%) (x = 0, 3, 5, 10 mol%). (**b**) Galvanostatic charge–discharge curves for PANI and PANI (TA3%) recorded at a current density of 0.1, 0.3, 0.5 and 1.0 A g^−1^.

Consistent with the porosity–conductivity trade-off, we used cyclic voltammetry (CV) to identify the optimal TA content for capacitive performance. As shown in Figure 5a, PANI (TA 3%) exhibits the highest CV current, featuring a redox-dominant capacitive profile with distinct peaks centered at ~0.62 V and ~0.71 V (vs. Ag/AgCl), corresponding to the transitions between the emeraldine and pernigraniline states of polyaniline [31]. This redox activity, characteristic of pseudocapacitive materials, contributes to enhanced charge storage. Specific capacitances (Table 1) reach 556 F g^−1^ for PANI (TA 3%) versus 398 F g^−1^ for pristine PANI. Higher TA loadings reduce performance (530 F g^−1^ at 5% and 40 F g^−1^ at 10%), with the decline at 5% attributed to lower conductivity despite higher porosity.

Device-level performance was evaluated by galvanostatic charge–discharge (GCD) in a Swagelok cell (Figure 5b; Table S1). At 0.1 A g^−1^, PANI (TA 3%) delivers longer discharge times and less-distorted profiles, corresponding to 239 F g^−1^—substantially higher than pristine PANI (199 F g^−1^). At 1 A g^−1^, the capacitance decreases to 197 F g^−1^, reflecting partial transport limitations under high-rate conditions.

To further elucidate the rate capability, we compared the structural influence on charge–discharge behavior across current densities. At low rates, capacitive behavior is primarily governed by accessible electroactive surface area. In this regime, the roughened and hierarchically porous structure of PANI (TA 3%) provides abundant ion-accessible sites, enabling greater ion accumulation and higher apparent capacitance [32]. However, at high rates (≥1 A g^−1^), ion diffusion becomes rate-limiting, as the increased tortuosity of the mesoporous network and reduced electronic conductivity hinder rapid ion/electron transport [33]. In contrast, pristine PANI, possessing predominantly macroporous channels and higher intrinsic conductivity, facilitates more efficient charge transfer and ion diffusion, sustaining relatively higher capacitance retention at fast charge–discharge rates [34]. Therefore, the difference in rate capability between samples arises from a fundamental trade-off between surface area (ion storage capacity) and ion/electron transport efficiency. This behavior aligns with commonly observed trends in mesostructured pseudocapacitors, where excessive porosity enhances low-rate capacitance but compromises high-rate response due to extended ion migration pathways.

## 4. Conclusions

We developed hierarchically porous PANI by incorporating tetrakis(4-aminophenyl)methane (TA) as a tetrahedral branching monomer, which promotes concurrent star-shaped and linear chain growth during polymerization. Spectroscopy and diffraction indicate modest shortening of conjugation and relaxed packing with TA, while imaging and porosity analyses show increased mesoporosity and surface roughness. This establishes a tunable porosity–conductivity trade-off governed by TA loading.

An intermediate TA level best balances ion access and charge transport: the 3 mol% composition shows the most ideal CV response and delivers 239 F g^−1^ at 0.1 A g^−1^ (~20% over pristine PANI) with reasonable rate capability. Higher TA contents further enhance porosity but can impede electronic pathways, tempering high-rate performance.

The approach is direct and scalable, creating branched, porous architectures in one step with minimal processing. By adjusting TA concentration, crystallinity and surface accessibility can be co-optimized for target applications. For practical deployment, moderate TA loadings (≈2–4 mol%) are recommended for energy storage electrodes, whereas high-power devices may benefit from lower TA contents or hybrid designs incorporating conductive additives.

## Figures and Tables

**Table 1:** BET surface area, BJH average pore diameter, electrical conductivity, and specific capacitance of PANI and PANI (TA-x%).

Sample	BET Surface Area (m^2^/g)	Average Pore Diameter (Nm)	Conductivity (S/cm)	Specific Capacitance (F/g)
PANI	45	73	1.23	398
PANI (TA 3%)	77	48	0.30	556
PANI (TA 5%)	95	45	0.032	530
PANI (TA 10%)	77	48	1.02 × 10^−6^	40

## Data Availability

The original contributions in this study are included in the article/Supplementary Materials. Further inquiries can be directed to the corresponding author.

## Supplementary Materials

The following supporting information can be downloaded at https://www.mdpi.com/article/10.3390/polym17223062/s1, Figure S1: Cumulative surface areas of PANI and PANI (TA x%), Figure S2: Schematic of TA-assisted aniline polymerization and packing model, Figure S3: Color evolution photographs of the early-stage reaction mixture, Figure S4: SEM image of PANI (TA 20%), Figure S5: GCD curves at 0.1 A g^−1^ (PANI; PANI (TA 3%)), Figure S6: GCD curves at 1 A g^−1^ (PANI; PANI (TA 3%)), Table S1: Specific capacitances and current densities from GCD.

## Abbreviations

The following abbreviations are used in this manuscript:

TA	Tetrakis(4-aminophenyl)methane
APS	Ammonium Persulfate
PANI	Polyaniline
PANI(TA x%)	Polyaniline from copolymerization of aniline and TA (x mol% relative to aniline)
