# Supplementary material for: Hierarchically Porous Polyaniline Exhibiting Enhanced Pseudocapacitive Property from Copolymerization of Aniline and Tetrakis(4-aminophenyl)methane
Source: Polymers (Basel). 2025 Nov 19;17(22):3062. doi: 10.3390/polym17223062 (PMC12656649; doi:10.3390/polym17223062)
Supplement: Supplementary file 1 [file polymers-17-03062-s001.zip › polymers-3950397-supplementary.pdf]

Supporting Information

Hierarchically Porous Polyaniline Exhibiting Enhanced Pseudocapacitive Property from Copolymerization of Aniline and Tetrakis(4-aminophenyl)methane

Jinsoon Choi, Kyeong Eun Yeo and Ji-Woong Park *

Department of Materials Science and Engineering, Gwangju Institute of Science and Technology (GIST), 123 Cheomdangwagiro, Gwangju 61005, Republic of Korea; cjs890221@gm.gist.ac.kr (J.C.); keyeo1462@gm.gist.ac.kr (K.E.Y.)

* Correspondence: jiwoong@gist.ac.kr

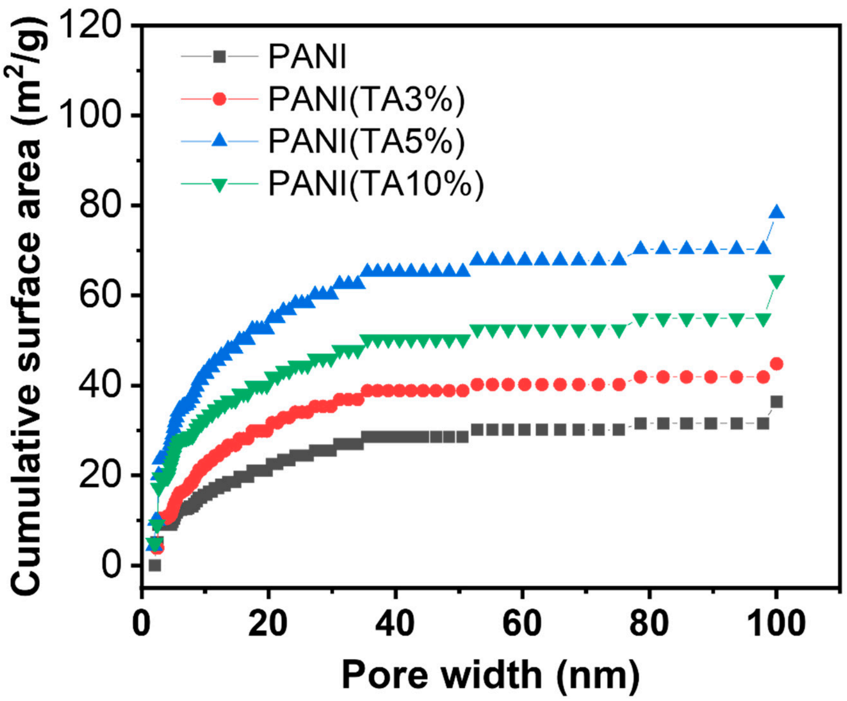

Figure S1. Cumulative surface areas of PANI and PANI (TA x%)

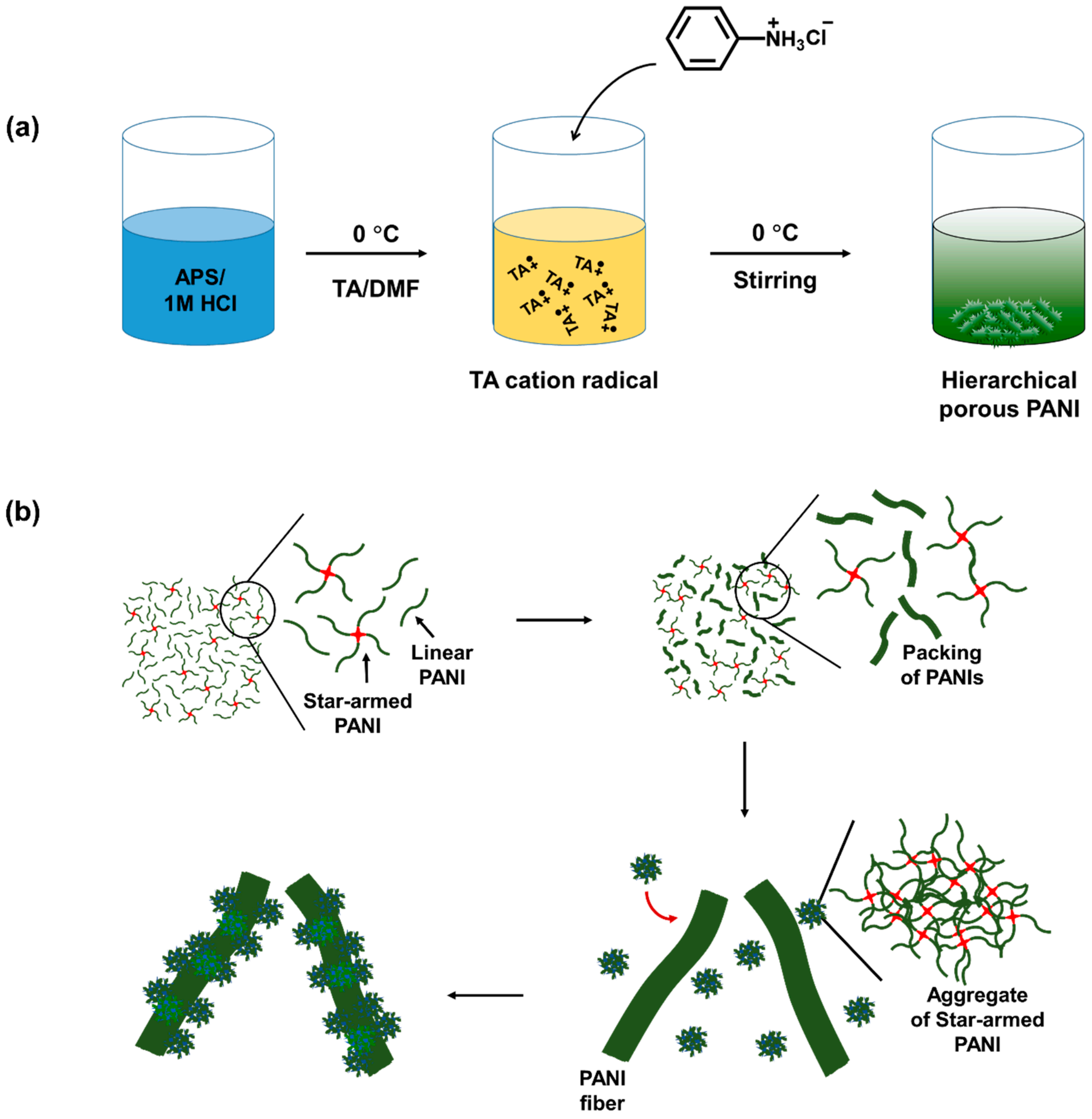

Figure S2. Formation pathway and tentative packing/crystallization model for TA-assisted aniline polymerization.

(a) Two-step protocol: TA is pre-oxidized with APS in 1 M HCl to generate transient TA-derived oxidized species; subsequent aniline addition under the same acidic/oxidative conditions yields PANI.

(b) Tentative mechanism of simultaneous chain formation and packing. Linear PANI and TA-derived "star-armed" PANI form concurrently, and their packing/crystallization also proceeds concurrently. Owing to the steric hindrance imparted by TA, star-armed chains crystallize less readily and tend to aggregate. These star-armed aggregates adhere to/decorate the growing crystallites or packed bundles of linear PANI, producing a "sushi-kebab-like" architecture (linear PANI as the skewer; star-armed PANI aggregates as the toppings) and leading to a hierarchically porous network.

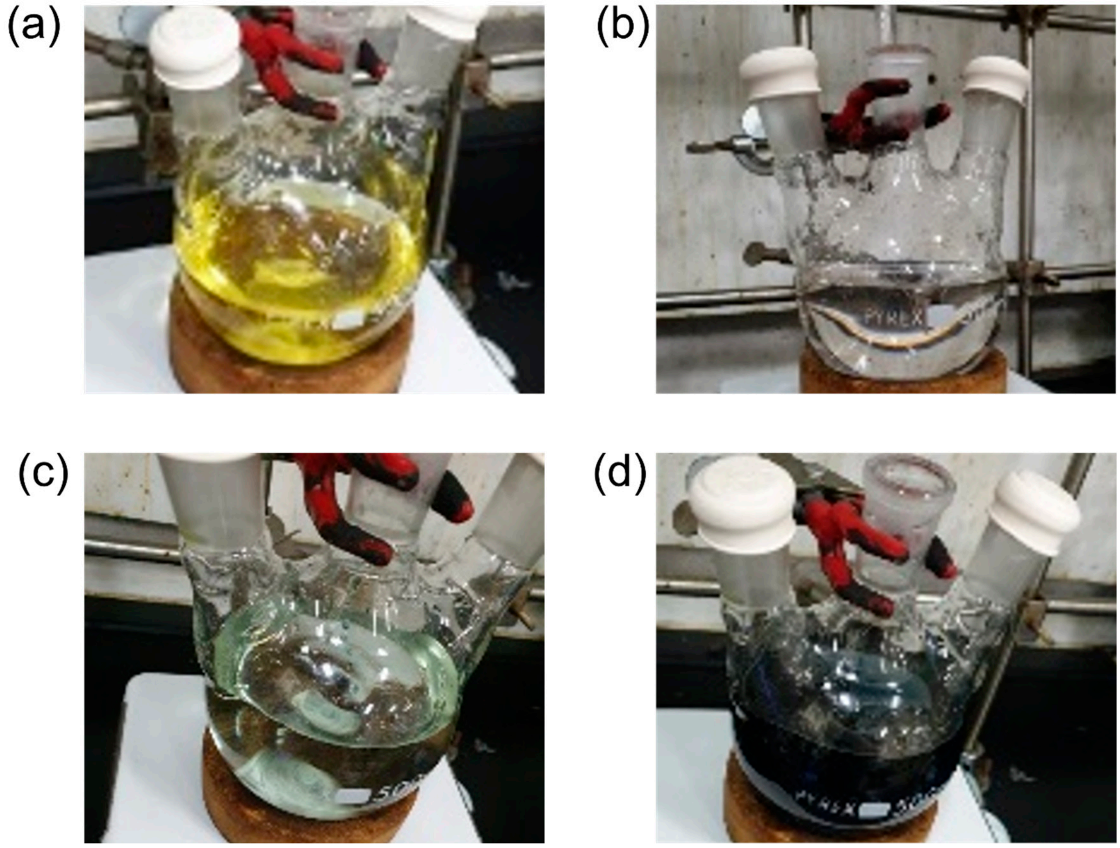

Figure S3. Photographic sequence showing the color evolution of the reaction mixture during the early-stage oxidative copolymerization of aniline and TA (tetrakis(4-aminophenyl)methane) in 1 M HCl at 0–5 °C. (a) Upon addition of APS to the TA solution, the colorless solution turned yellow, indicating the formation of TA radical cations. (b) Dropwise addition of aniline dissolved in 1 M HCl led to rapid decolorization, reflecting consumption of TA radical cation via coupling with anilinium radical cation. (c) Within ~30 minutes, the solution gradually turned pale green as polymerization initiated. (d) Continued reaction for more than 6 hours resulted in the formation of a dark bluish-green viscous mixture, characteristic of growing polyaniline chains with mixed oxidation states.

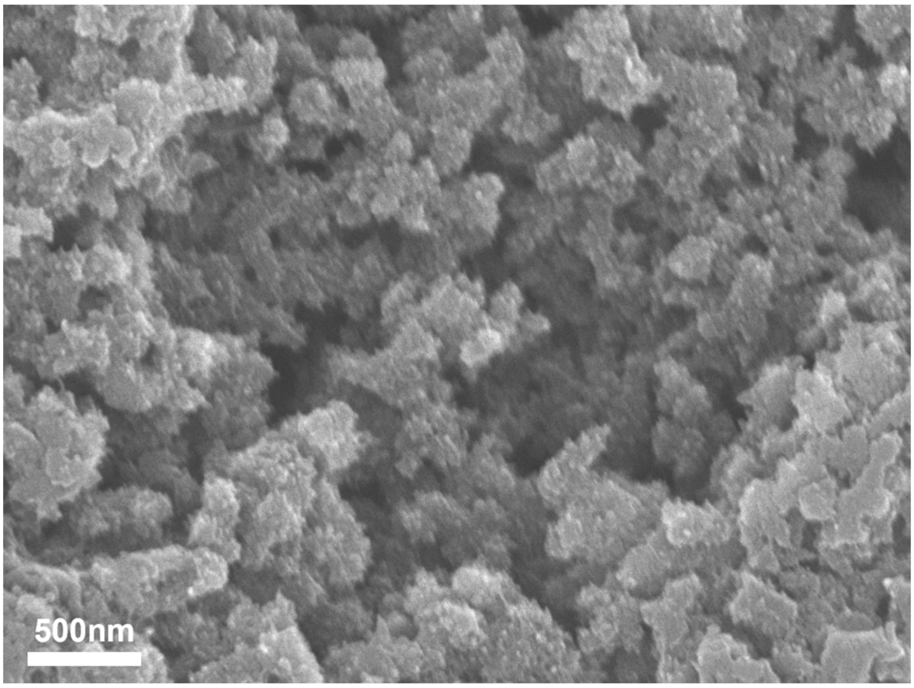

Figure S4. SEM image of PANI (TA 20%). The sample shows irregular, aggregated granular domains with no discernible nanofiber network, consistent with nanofiber formation being hindered at high TA loading and promoting dense particle aggregation.

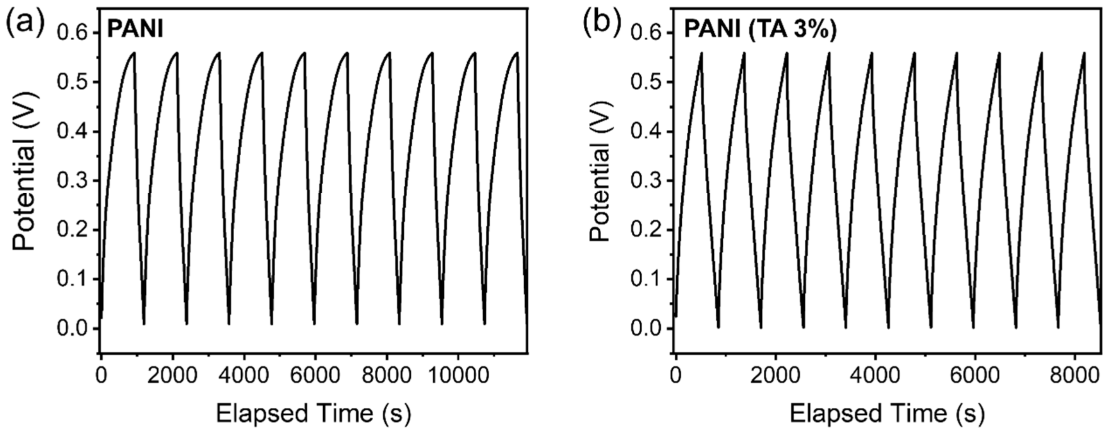

Figure S5. Galvanostatic charge–discharge (GCD) curves recorded at a current density of 0.1 A g^{-1} for 10 consecutive cycles. (a) PANI (b) PANI (TA 3%).

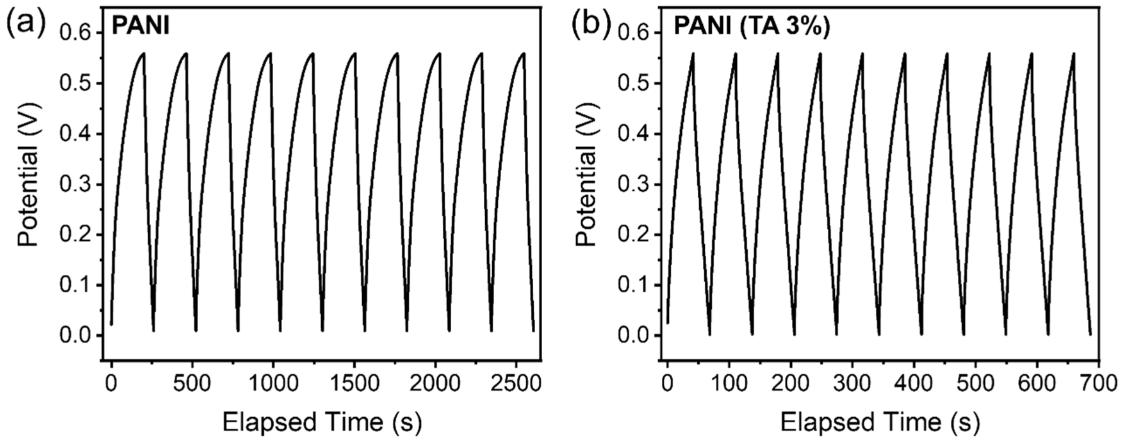

Figure S6. Galvanostatic charge–discharge (GCD) curves recorded at a current density of 1 A g^{-1} for 10 consecutive cycles. (a) PANI (b) PANI (TA 3%).

Table S1. Specific capacitances of PANI and PANI(TA3%) with current density calculated from GCD measurement.

Current density	PANI<br>[F/g]	PANI(TA3%)<br>[F/g]
0.1A/g	199	239
0.2A/g	206	242
0.5A/g	480	233
